# Serum Lactate-Albumin Ratio: Soothsayer for Outcome in Sepsis

**DOI:** 10.7759/cureus.36816

**Published:** 2023-03-28

**Authors:** Ruchita Kabra, Sourya Acharya, Samarth Shukla, Sunil Kumar, Anil Wanjari, Satish Mahajan, Shilpa A Gaidhane, Pratik J Bhansali, Praful Wasnik

**Affiliations:** 1 Department of Internal Medicine, Jawaharlal Nehru Medical College, Datta Meghe Institute of Higher Education & Research, Wardha, IND; 2 Department of Pathology, Jawaharlal Nehru Medical College, Datta Meghe Institute of Higher Education & Research, Wardha, IND; 3 Department of Radiodiagnosis, Jawaharlal Nehru Medical College, Datta Meghe Institute of Higher Education & Research, Wardha, IND

**Keywords:** albumin, lactate, lactate to albumin ratio, sofa, sepsis

## Abstract

Aim: The aim of this study is to assess the efficacy of the serum lactate/albumin (L/A) ratio as a prognostic marker of sepsis syndrome.

Materials and methods: This study was conducted in the Internal Medicine Department at Acharya Vinoba Bhave Rural Hospital with a sample size of 160 cases of sepsis. The serum L/A ratio was calculated on admission and correlated with deaths and morbidity. Statistical analysis was significant if the P-value was less than 0.05.

Results: The mean age of patients was 52.83 ± 16.80 years with a male predominance (64.4% vs. 35.6%). The mean L/A ratio was 0.95 ± 0.46. The proportion of discharged subjects and mortality were 58.8% and 41.2%, respectively. The study found that a higher mean L/A ratio (1.1-1.44) was significantly linked to the various variables in the study. Furthermore, a significantly higher median L/A ratio of 1.23 was found in subjects with vasopressor use. The median L/A ratio in the Discharge group and Death group was 0.64 and 1.27, respectively. The area under the receiver operating characteristic (AUROC) curve indicated that accurate diagnostic performance was 0.976 in predicting Death versus Discharge for the L/A ratio.

Conclusion: This study found that, compared to lactate and albumin alone, the predictor value of the L/A ratio was outstanding in predicting death and hospital stay (discharge) among sepsis participants, with a sensitivity of 100% and a specificity of 88%.

## Introduction

Sepsis is a life-threatening medical condition in which the body’s immune system reacts in such a way to any infections, potentially leading to the malfunctioning of various organs, shock, and death [1]. Sepsis remains the leading cause of morbidity and death in patients who have had a prolonged intensive care unit (ICU) stay or are critically ill globally. The incidence and prevalence of mortality rates due to severe sepsis are commonly underrated. The global prevalence of sepsis was 31.5 million cases, with 5.3 million deaths annually [2]. Sepsis leads to mortality in the range of 20%-50%, of which 35% contributes to inhospital deaths [3,4]. According to the World Health Organization (WHO), the overall load of sepsis is difficult to determine, but a recent scientific paper evaluated deaths due to sepsis, i.e., in 2017, 11 million deaths out of 48.9 million cases globally, which were considered 20% of overall deaths [5]. The fatality rate in India is found to be 213 per 1,00,000 patients with the diagnosis of sepsis [6].

Sepsis occurs due to inflammation, which results in platelets triggering endothelial cell destruction. When microbial substances activate Toll-like receptors, which results in multiple organ failure, shock, and eventual death, sepsis is caused. According to the most recent research, both endogenous chemicals and microorganisms can activate Toll-like receptors [7]. For the rapid establishment of the site of infection, its pathogen, and management in the form of aggressive fluid resuscitation, the risk stratification of sepsis is also based on clinical signs and laboratory findings. Important investigations and markers for early diagnosis, management, risk stratification, and prognosis of sepsis/severe sepsis include total leucocyte counts, platelet counts, blood sugar, serum lactate levels, serum albumin levels, C-reactive protein, procalcitonin, blood culture, and urine culture [8].

Lactic acidosis occurs as a result of cellular dysfunction, tissue hypoperfusion, and increased aerobic glycolysis in sepsis and severe sepsis [9]. In severely ill patients, hypoalbuminemia indicates the severity of the inflammation taking place. Although serum lactate and serum albumin have individual predictive values for mortality in sepsis and critically ill patients, the combined effect of the serum lactate/albumin (L/A) ratio is a better predictor. The L/A ratio has shown better results in identifying high-risk patients and preventing deaths. Wang et al. also concluded that an increased L/A ratio is linked with mortality in sepsis patients [10]. The Sequential Organ Failure Assessment (SOFA) score was used for predicting deaths in patients with sepsis in the ICU. A score of two or more was considered a predictor of mortality in sepsis patients [11-13].

The major key parameters in the treatment of sepsis are early recognition and timely administration of broad-spectrum antibiotics [10,14]. Delay in aggressive treatment and management is connected to very high morbidity and mortality rates. Despite recent evolution in the awareness and understanding of the etiopathogenesis of sepsis and advancements in various tools of sepsis management, sepsis remains the major cause of death as well as morbidity in severely ill patients.

This study determined various values of L/A ratio in different classes of sepsis, which helped to study the patient outcome in terms of the need of ventilatory support, the need of vasopressors, long duration of hospital stay, recovery, and deaths.

## Materials and methods

This is a cross-sectional study that was done at a rural hospital in Central India over a two-year period from December 2020 to December 2022. Ethical approval was obtained from the Institutional Ethics Committee of Datta Meghe Institute of Medical Sciences [Reference no.: DMIMS(DU)/IEC/2020-21/9289]. The population of the study was the cases of sepsis as diagnosed by the Third International Consensus Definitions for Sepsis and Septic Shock (SEPSIS-3) criteria [15].

According to Chatterjee et al. [16], the required sample size was 160, depending on the prevalence of 28.3%. This study included patients over the age of 18 who were admitted to the Medicine ICU and diagnosed with sepsis using SEPSIS-3 criteria. Patients with known cases of liver cirrhosis, nephrotic syndrome, and chronic kidney disease were excluded from the study, as these conditions are associated with hypoalbuminemia.

**Figure 1:**
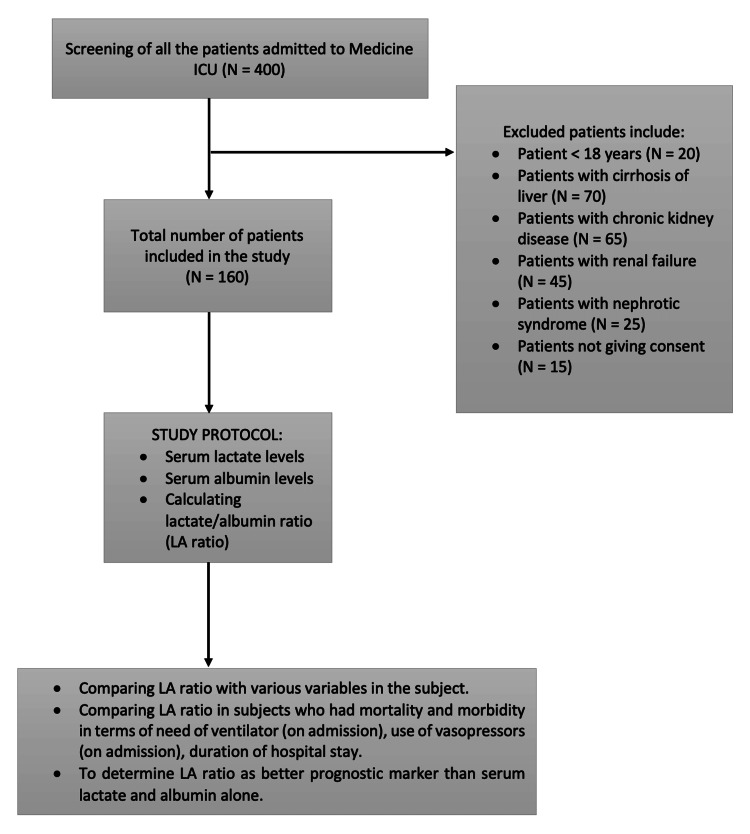
Flowchart for the study procedure. LA (L/A): lactate/albumin.

Subjects were screened and explained the study procedure in their native language in detail. The study group consisted of subjects who were willing to participate, and the consent document was signed by the subject himself/his relatives. Figure 1 shows the flowchart for the study procedure.

A thorough history was taken, a physical examination was performed on all the patients, and laboratory testing was performed, which included complete blood count (CBC): hemoglobin, platelet count, white blood cells, albumin, urea, creatinine, lactate, L/A ratio, serum glutamic oxaloacetic transaminase (SGOT), serum glutamic pyruvic transaminase (SGPT), bilirubin, and glucose levels. The normal limit of serum lactate was 0.7-2.1 mmol/L and serum albumin was 3.5-5.0 g/dL. The serum L/A ratio was calculated.

Other details obtained from the patients included their age, gender, and comorbidities such as diabetes mellitus and hypertension. Chronic obstructive pulmonary disease (COPD) and stroke were both combined in other comorbidity categories. The patient’s sensorium in the form of the Glasgow Coma Scale (GCS) and vital signs were evaluated. Also, the need of ventilatory support and the need of vasopressors at the time of admission were recorded. The effectiveness of the serum L/A ratio as a predictive determinant for sepsis syndrome in comparison with serum lactate and serum albumin alone was evaluated statistically. Statistical analysis was done such that outcome (death vs. discharge) was considered as the primary outcome variable. Lactate, albumin, and L/A ratio were considered as secondary outcome variables. Baseline characteristics such as age, comorbidities, and blood investigations, such as total leukocyte count (TLC) (/mm³), platelet count (Lacs/mm³), urea (mg/dL), albumin (g/dL), creatinine (mg/dL), bilirubin (mg/dL), SGPT (U/L), lactate (mmol/L), SGOT (U/L), SOFA score, and alkaline phosphatase (U/L), were considered as primary explanatory variables. For quantitative variables, the mean and standard deviation (SD) were used in the descriptive analysis, while frequency and proportion were used for categorical variables. Mann-Whitney U test was used for medians and the interquartile range (IQR) for quantitative parameters with aberrant distribution was compared between research groups (two groups). A chi-square test was used for categorical outcomes. Receiver operating characteristic (ROC) analysis was performed for lactate, albumin, and L/A ratio. The relevant area under the curve (AUC) and cutoff values are defined based on sensitivity and specificity along with diagnostic accuracy tabulated for all the variables. A significant study was considered if the P-value was lower than 0.05.

## Results

Of 160 patients enrolled in the study, 62 (38.7%) were in the age group 41-60 years, with a male predominance of 64.4%. Among all, 27.5% of patients had infections such as COVID-19, dengue, and malaria; 21.9% had respiratory infections such as pneumonia and other respiratory disorders; 15% had neurological infections such as meningitis, encephalitis, and other neurological diseases; 11.2% were affected by cardiovascular infections such as infective endocarditis, myocarditis, and other cardiovascular diseases; and others include skin infections and sepsis due to poisoning (Table 1).

**Table 1:** Descriptive analysis for baseline qualitative characteristics (N = 160). COPD: chronic obstructive pulmonary disease.

Basic details	Frequency (%)
Age (years)	
18-40	40 (25)
41-60	62 (38.7)
>60	58 (36.2)
Sex	
Female	57 (35.6)
Male	103 (64.4)
System involved	
Infectious	44 (27.5)
Respiratory	35 (21.9)
Neurology	24 (15.0)
Cardiology	18 (11.2)
Gastroenterology	12 (7.5)
Renal	17 (10.6)
Others	10 (6.2)
Comorbidity	Yes
Hypertension	53 (33.1)
Diabetes mellitus	33 (20.6)
Others (COPD, stroke)	33 (20.6)
Need of ventilator (admission) (yes)	38 (23.8)
Vasopressor use (admission) (yes)	56 (35.0)
Study parameters	Frequency (%)
Lactate (on admission)	
≤2.1 mmol/L	57 (35.6)
>2.1 mmol/L	103 (64.4)
Albumin	
<3.5 g/dL	125 (78.1)
≥3.5 g/dL	35 (21.9)
Length of stay	
≤14 Days	118 (73.8)
>14 Days	42 (26.2)
Outcome	
Discharge	94 (58.8)
Death	66 (41.2)

The variables that had a significant relationship (P < 0.05) with L/A ratio are heart rate (/min), respiratory rate (/min), systolic BP (mmHg), O_2_ saturation, PaO_2_/FiO_2_, need of ventilator (admission), vasopressor use (admission), TLC (/mm³), platelet count (Lacs/mm³), urea (mg/dL), albumin (g/dL), creatinine (mg/dL), bilirubin (mg/dL), SGPT (U/L), lactate (mmol/L), SGOT (U/L), SOFA score, alkaline phosphatase (U/L), and outcome, as described in Table 2.

**Table 2:** Association of baseline parameters with L/A ratio (N = 160). L/A: lactate/albumin; SOFA: Sequential Organ Failure Assessment; SGOT: serum glutamic oxaloacetic transaminase; SGPT: serum glutamic pyruvic transaminase; RBS: random blood sugar; TLC: total leucocyte count. 1: Spearman correlation, 2: Kruskal-Wallis test, 3: Wilcoxon-Mann-Whitney U test. ***Significant at P < 0.05.

Parameters	L/A ratio	P-value
Age (years)	Correlation coefficient (rho) = 0.09	0.233^1^
Age		0.655^2^
18-30	0.77 ± 0.36	
31-40	0.93 ± 0.30	
41-50	0.99 ± 0.50	
51-60	0.92 ± 0.49	
61-70	1.03 ± 0.50	
71-80	1.03 ± 0.54	
81-90	0.87 ± 0.49	
Gender		0.317^3^
Male	0.99 ± 0.50	
Female	0.88 ± 0.38	
System involved		0.539^2^
Infectious	0.96 ± 0.41	
Respiratory	1.00 ± 0.51	
Neurology	0.94 ± 0.41	
Cardiology	0.92 ± 0.50	
Gastroenterology	0.79 ± 0.33	
Renal	1.01 ± 0.64	
Others	1.18 ± 0.66	
Heart rate (/min)***	0.68	<0.001^1^
Respiratory rate (/minutes)***	0.64	<0.001^1^
Systolic blood pressure (mmHg)***	-0.54	<0.001^1^
O_2_ saturation***	-0.67	<0.001^1^
Need of ventilator (admission)***		<0.001^3^
Yes	1.31 ± 0.30	
No	0.84 ± 0.45	
Vasopressor use (admission)***		<0.001^3^
Yes	1.29 ± 0.50	
No	0.77 ± 0.32	
	Correlation Coefficient (rho)	
Hemoglobin	-0.08	0.341^1^
TLC ***	0.38	<0.001^1^
Platelet count ***	-0.52	<0.001^1^
SOFA score***	0.76	<0.001^1^
SGOT***	0.37	<0.001^1^
SGPT***	0.36	<0.001^1^
Alkaline phosphatase***	0.2	0.010^1^
Globulin	-0.08	0.319^1^
Bilirubin***	0.47	<0.001^1^
Urea***	0.42	<0.001^1^
Creatinine***	0.47	<0.001^1^
Sodium	0.06	0.441^1^
Potassium	0.1	0.217^1^
RBS	0.04	0.657^1^
Lactate***		<0.001^3^
≤2.1 mmol/L	0.54 ± 0.15	
>2.1 mmol/L	1.18 ± 0.42	
Albumin***		<0.001^3^
<3.5 g/dL	1.06 ± 0.45	
≥3.5 g/dL	0.54 ± 0.20	
Length of stay		0.792^3^
≤14 Days	0.95 ± 0.49	
>14 Days	0.93 ± 0.37	
Outcome***		<0.001^3^
Death	1.37 ± 0.38	
Discharge	0.65 ± 0.23	

The comparison of the L/A ratio with the need of ventilator on admission showed a median L/A ratio of 1.26 (1.1-1.44) and 0.72 (0.51-1.06) in the patients who did not have a need of ventilator on admission. The median L/A ratio was higher in patients who required a ventilator (admission), and the two groups showed a significant difference (W = 3927.000, P = 0.001).

**Figure 2:**
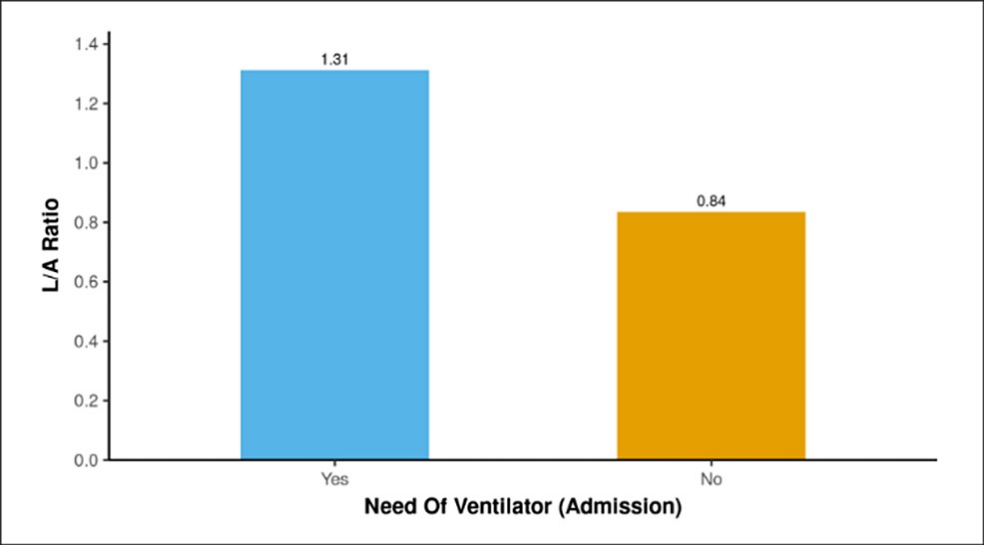
Association between the need of ventilator (admission) and L/A ratio. L/A: lactate/albumin.

The bar graph shown in Figure 2 describes the mean L/A ratio in the two groups needing ventilator support on admission.

The median L/A ratio was 1.23 (1.05-1.45) in the cases that used vasopressors on admission, and it was 0.72 (0.5-1.01) in the non-vasopressor group. The median L/A ratio was highest in the vasopressor use (admission) group, and the two groups showed a significant difference (W = 4779.500, P = 0.001).

**Figure 3:**
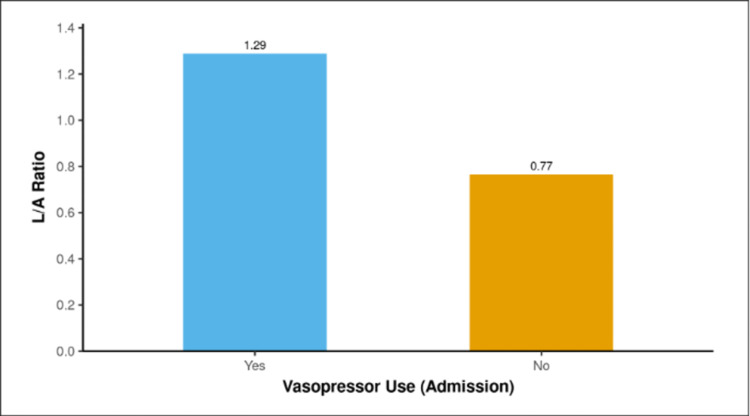
Analysis of the association between vasopressor use (admission) to L/A ratio. L/A: lactate/albumin.

The mean L/A ratio for the two groups of patients who used vasopressors at the time of admission is displayed in the bar graph shown in Figure 3.

**Figure 4:**
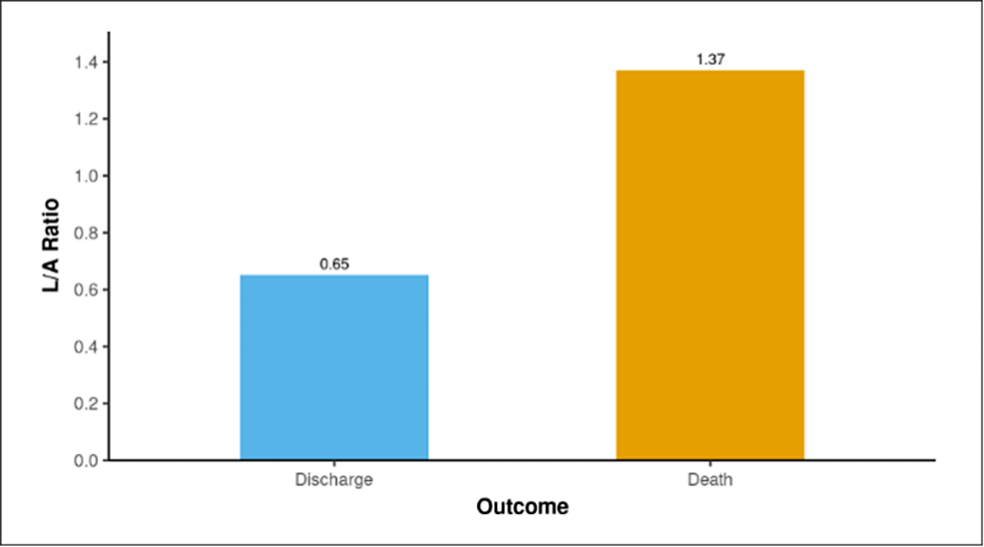
Association between outcome and L/A ratio. L/A: lactate/albumin.

The median L/A ratio was 0.64 (0.48-0.78) in the discharged cases and 1.27 (1.11-1.46) in the mortality cases. There was a significant difference in the L/A ratio between death and discharged cases (W = 146.500, P ≤ 0.001), with the median L/A ratio being maximum in the death group. The mean L/A ratio for the two groups, Discharge and Death, is displayed in the bar graph shown in Figure 4.

**Figure 5:**
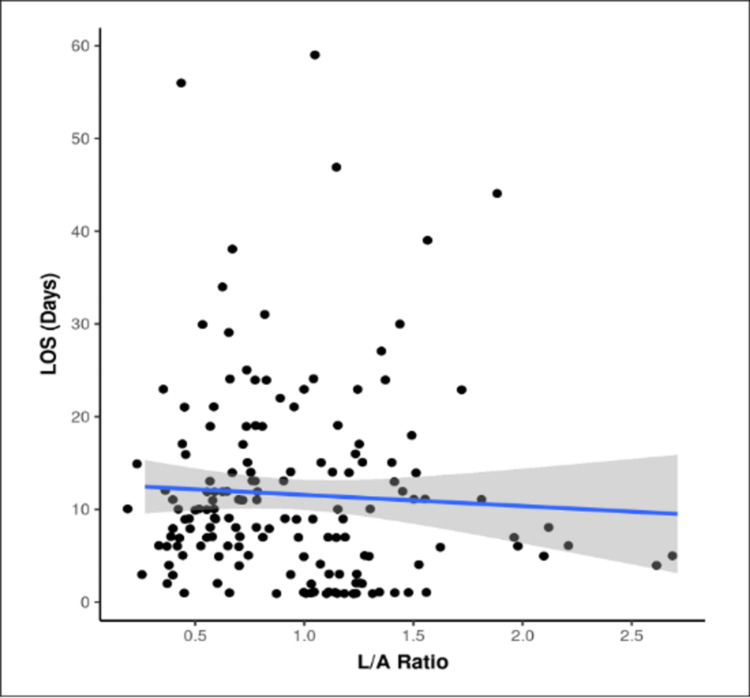
Scatter diagram for LOS and L/A ratio (N = 160). LOS: length of stay; L/A: lactate/albumin.

The two subgroups of the length of stay (LOS) for the variable L/A ratio did not have a normal distribution. The median L/A ratio obtained in the LOS ≤14 days group was 0.91 (0.58-1.24) and in the LOS >14 days group was 0.83 (0.66-1.22). Among the two groups of ≤14 days and >14 days, the difference was not significant for L/A ratio (W = 2409.500, P = 0.792). The scatterplot in Figure 5 suggests the correlation between L/A ratio and LOS (Days). Each point represents a distinct case. The overall pattern of correlation is depicted by the blue trendline. The confidence interval of this trendline is shown by the gray-shaded area.

**Figure 6:**
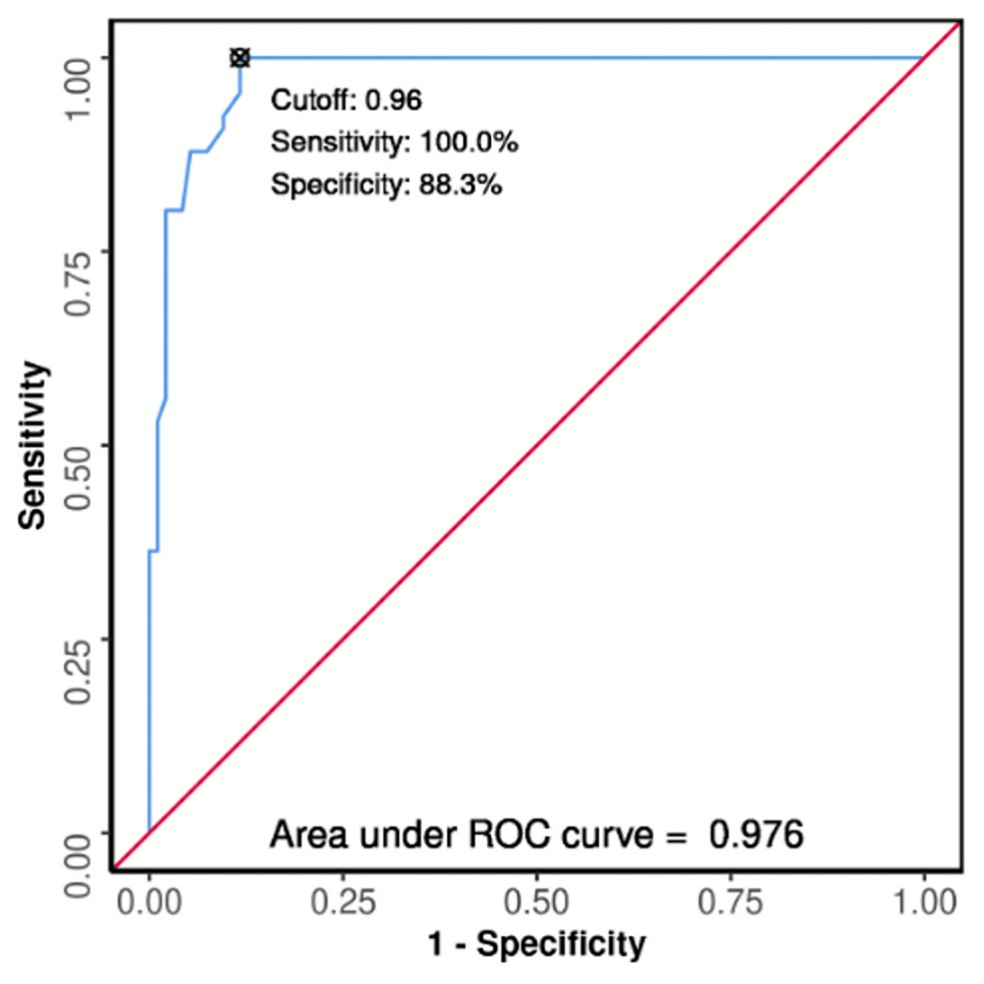
ROC curve analysis demonstrating the L/A ratio's diagnostic performance in predicting death versus discharge (N = 160). L/A: lactate/albumin; ROC: receiver operating characteristic.

The excellent diagnostic performance of the L/A ratio forecasting Death versus Discharge outcome was 0.976 (95% CI: 0.957-0.996) (P ≤ 0.001), which was suggestive of statistical significance. The cutoff of L/A ratio ≥0.96 has a sensitivity of 100% and a specificity of 88% in predicting Death as depicted in Figure 6.

**Figure 7:**
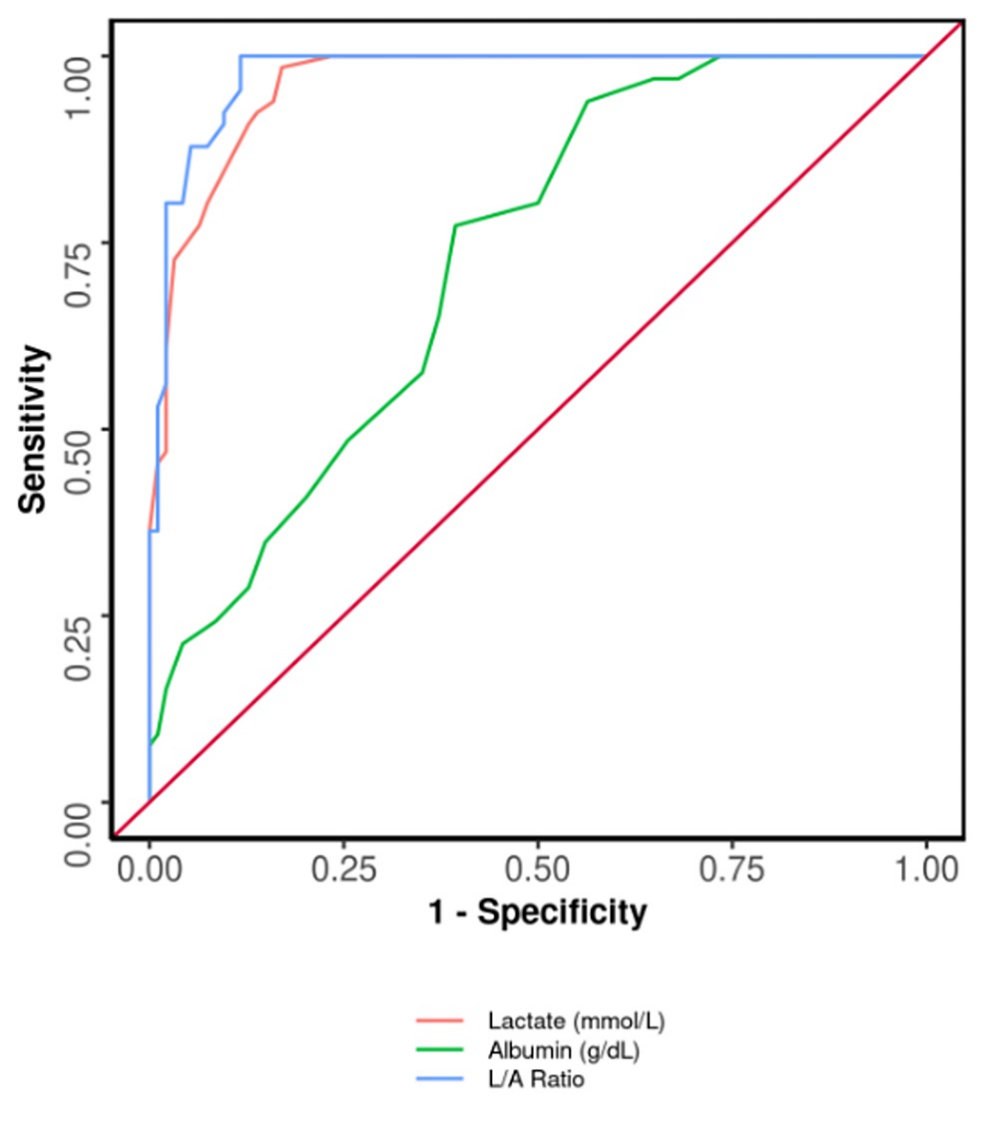
ROC curve analysis demonstrating diagnostic performance for L/A ratio, lactate, and albumin in forecasting death versus discharge (N = 160). L/A: lactate/albumin; ROC: receiver operating characteristic.

The area under the ROC (AUROC) curve showing a comparison among albumin, lactate alone, and L/A ratio is shown in Figure 7. Parameters like L/A ratio, lactate (mmol/L), and albumin (g/dL) significantly predicted the outcome: death. Also, the ideal variable for AUROC was L/A ratio. With respect to sensitivity, specificity, positive predictive value, negative predictive value, and diagnostic accuracy, the L/A ratio was the best parameter. The diagnostic accuracy of the L/A ratio and lactate in predicting mortality was 93% and 89%, respectively. Lactate alone is also a predictor of mortality, but L/A Ratio is a better predictor marker.

## Discussion

Sepsis continues to have a high rate of morbidity and death, according to recent research conducted globally. According to these studies, more than 30 million individuals worldwide suffer from sepsis each year. Sepsis affects 1%-2% of hospital inpatients each year [17]. Roughly 15% of these patients will develop septic shock, which happens in 10% of ICU admissions with a death rate of 50% [17]. A retrospective study that included 419 sepsis patients revealed that the mortality rate for patients in the ICU of their teaching hospital was as high as 43.9 [18]. According to an epidemiological survey conducted in China, patients with sepsis had a mortality rate of 48.7% [18].

Blood lactate levels can rise owing to tissue hypoxia-induced anaerobic metabolism or poor liver clearance [19,20]. Multiple studies have shown that increased lactate is closely linked with the outcome of the patient [21,22]. Hence, the present study showed that sepsis and severe sepsis patients had variable levels of L/A ratio. In addition, the outcome was assessed in terms of the need of ventilatory support, the need of vasopressors, recovery, and mortality in comparison with the L/A ratio in patients with sepsis and severe sepsis. This cross-sectional study included 160 subjects with a mean age of 52.83 ± 16.80 years. There was a male predominance in our study population (64.4% vs 35.6%). The proportion of discharged subjects was 58.8% and mortality was 41.2%.

Various etiologies of sepsis in these patients were studied, of which the most common cause was infections. Infections include COVID-19 infection, dengue, scrub typhus, malaria, and HIV/AIDS. Out of 160 patients, 44 had infections (27.5%). Other systems were also involved. Thirty-five of 160 had respiratory causes (21.9%), 24 of 160 had neurological causes (15%), 18 of 160 had cardiological causes (11.2%), 17 of 160 had renal causes (10.6%), 12 of 160 had gastrointestinal causes (7.5%), and others 10 of 160 accounted of 6.2%. 

Depending on the cutoff of serum lactate, the study population was grouped as ≤2.1 mmol/L in 35.6% and >2.1 mmol/L in 64.4%. The mean heart rate, respiratory rate, systolic BP, oxygen saturation, PaO_2_/FiO_2_, need of ventilator, vasopressor use, TLC, platelet count, SOFA score, SGOT, SGPT, bilirubin, creatinine, urea, lactate, albumin, and L/A ratio were found to be significantly (P < 0.05) greater in the subjects with serum lactate levels >2.1 mmol/L in comparison with individuals with serum lactate levels ≤2.1 mmol/L. In addition, the mortality rate was high in patients with serum lactate levels significantly >2.1 mmol/L, whereas there was no mortality in those with serum lactate levels ≤2.1 mmol/L. Vasopressor use and the need of ventilation were high in the group where lactate was >2.1, which were 47 (45.6%) and 38 (36.9%), respectively.

The significant rise in lactate testing usage at our hospitals is probably the result of a number of factors. The predictive value of lactate [23], its use as a therapeutic goal for resuscitation, its use for aggressive management, and the promise of quick testing to enhance patient outcomes. A prospective study by Shapiro et al. [24] involving 1278 subjects observed that as the lactate levels increased, the mortality rate increased (between 0 and 2.5 mmol/l, 4.9% death; 2.5 and 4.0 mmol/L, 9% death; ≥4.0 mmol/L, 28.4%). Shapiro et al. [24] recorded the lactate cutoff of more than 4.0 mmol/L having 92% specificity and 36% sensitivity for any death, and for death within three days, it was 55% sensitive and 91% specific. Another similar study by Hayashi et al. [25] with a cutoff of lactate 3.05 mmol/L had a sensitivity of 64.1% and specificity of 77.4% in foretelling hospital mortality. However, in our study, the sensitivity of lactate was 98.5% in predicting death, specificity was 83%, and diagnostic accuracy was 89.4%. In 279 emergency patients, Martín-Rodríguez et al. [26] reported that prehospital lactate levels were an effective indicator of mortality. Jansen et al. [21] narrated that lactate levels in pre-hospitalized 124 patients represented a significant relation to mortality. According to Bou Chebl et al. [27], high lactate groups were shown to have higher inhospital mortality rates and lengthier hospital stays. When assessed anywhere (ED, ICU, or general ward), initial lactate of 4.0 mmol/L was strongly associated with acute-phase mortality in infected patients (three days), according to Trzeciak et al. [28]. After controlling for confounders, Hayashi et al. [25] found no statistically significant results (P = 0.056) for the initial maximum lactate (max lactate at T0). Numerous studies showed that high serum lactate levels were related to poor outcomes [29,30].

Albumin <3.5 g/dL was found in 78.1% and ≥3.5 g/dL in 21.9%. The present study found that serum albumin <3.5 g/dL significantly (P < 0.05) showed marked variation with heart rate, respiratory rate, systolic BP, oxygen saturation, need of ventilator (admission), vasopressor use (admission), hemoglobin, TLC, platelet count, SOFA score, SGOT, globulin, lactate, bilirubin, creatinine, urea, albumin, lactate, and L/A ratio. Mortality was greater among the subjects with <3.5 g/dL serum albumin, which was 51.2%, and discharge was found in 48.8%. In addition, vasopressor usage and need of ventilation support were high in the albumin<3.5 group with 52 (41.6%) and 36 (28.8%), respectively.

Since blood albumin estimation is quick and the test is widely accessible, serum albumin is a more accurate indicator of malnutrition than body mass index (BMI). Serum albumin values of more than 3.5 g/dL are considered as an appropriate reserve of serum albumin. It offers protection from several organic processes that might be harmful. Numerous studies have demonstrated that patients with lower blood albumin levels (3.5 g/dL) had poor clinical outcomes [31]. Acute and chronic reduced levels of serum albumin have both been freely linked to a higher risk of mortality in sepsis. In individuals who develop catastrophic sepsis and organ failure, critical stage low blood albumin is connected to an increased risk of severity and death [32-34]. Low albumin levels were a risk factor for overall mortality in patients with acquired bloodstream infections who needed intensive care (OR, 0.34; 95% CI, 0.15-0.76) [35]. According to prior research by Vincent et al. [36], every 10 g/L of a substantial drop in serum albumin level escalated the risk for morbidity to 89%, death to 137%, long-term ICU stays by 28%, long-term hospital stays by 71%, and use of resource to 66%. Rathod et al. [37] reported in their study the predictive value of albumin done in the preoperative period for predicting mortality and morbidity.

The present study found that a higher mean ratio (1.1-1.44) was remarkably associated (P < 0.05) with 'L/A ratio': heart rate, respiratory rate, systolic BP, O_2_ saturation, PaO_2_/FiO_2_, need of ventilator (admission), vasopressor use (admission), TLC, platelet count, SOFA score, SGOT, bilirubin, SGPT, lactate, creatinine, albumin, urea, ALP, and outcome. Further, a significantly higher median L/A ratio of 1.23 was found in subjects with vasopressor use compared to non-vasopressor use (L/A ratio 0.72).

The mean (SD) L/A ratio in assessing the outcome of the Discharge group was 0.65 (0.23). The median L/A ratio for assessing the Discharge group was 0.64 (0.48-0.78). The median L/A ratio in the Death group was 1.27 (1.11-1.46). The AUROC for L/A ratio used in predicting death versus discharge has been 0.976 (95% CI: 0.957-0.996), which was signifying excellent diagnostic output. It showed statistical significance (P ≤ 0.001).

The specificity of L/A ratio in predicting death is 88%, whereas the specificity of lactate alone in predicting death is 83% and the specificity of albumin alone in predicting death is 61%. Also, the sensitivity of L/A ratio, lactate, and albumin in predicting the outcome is 100%, 98%, and 77%, respectively. The diagnostic accuracy of L/A ratio, albumin, and lactate in forecasting outcome is 93%, 68%, and 89%, respectively.

L/A ratio is a stronger predictive indicator in septic patients than lactate alone, according to the findings of prospective research by Bou Chebl et al. [38] (AUC of L/A ratio 0.65 vs. AUC of lactate 0.60), with a P-value of 0.001. Additionally, it showed that the L/A ratio has been linked to hospital deaths. The best cutoff value of L/A ratio, which distinguished non-survivors from survivors for all septic patients, was shown to be 0.115 [38]. Our study found a cutoff of L/A ratio ≥0.96, which predicts outcome: Death had a sensitivity of 100% and specificity of 88%. Makram et al. [39] reported that the L/A ratio was greater on days 0 and 1 in cases as compared to controls. Additionally, it was noticeably greater in patients who required ventilation compared to those who did not. Individuals who required renal replacement therapy (RRT) had a higher L/A ratio compared to those who did not. This study discovered that the L/A ratio was considerably greater in the non-survivors group as compared to survivors. The L/A ratio is highly effective than lactate alone in forecasting 28-day death, according to the biggest retrospective analysis conducted by Gharipour et al. [40] (AUC: 0.69 vs. 0.67, respectively). All these findings in this research are consistent with several studies examining the significance of the L/A ratio in a variety of illnesses, which also included sepsis in a prospective study of 155 patients, heart failure in a retrospective study of 4562 patients, and brain injury due to trauma in a retrospective study of 273 patients [41-43]. This study showed, that the L/A ratio was a reliable indicator for predicting deaths.

The limitation of this study was to validate the evaluation of the potential of the indices. Multicentered prospective studies with a large sample size are needed, and targeted studies on particular disease groups or systems involved are needed in the future.

## Conclusions

This cross-sectional study was done on a population of 160 patients in a rural setup. We found that albumin <3.5 g/dL, lactate ≥2.1 mmol/L, and L/A ratio predict mortality and need of ventilation, the need of vasopressor, and length of stay. The diagnostic accuracy of the L/A ratio was excellent in predicting mortality and hospital stay (discharge) among sepsis subjects as compared to lactate and albumin alone. A cutoff of L/A ratio ≥0.96 has a sensitivity of 100% and a specificity of 88% in predicting death. Calculating the L/A ratio in sepsis patients on admission can help in aggressive management and prevent mortality and morbidity. Easy availability and early results of the L/A ratio can help in assessing and predicting the outcome even at rural healthcare centers.
